# Amino acid metabolism as a therapeutic target in cancer: a review

**DOI:** 10.1007/s00726-021-03052-1

**Published:** 2021-07-22

**Authors:** Molly Endicott, Michael Jones, Jonathon Hull

**Affiliations:** Faculty of Health and Life Sciences, University of the West of England, Coldharbour Lane, Bristol, BS16 1QY UK

**Keywords:** Amino acids, Cancer, Oncology, Asparaginase, Metabolism

## Abstract

Malignant cells often demonstrate a proliferative advantage when compared to non-malignant cells. However, the rapid growth and metabolism required for survival can also highlight vulnerabilities specific to these malignant cells. One such vulnerability exhibited by cancer is an increased demand for amino acids (AAs), which often results in a dependency on exogenous sources of AAs or requires upregulation of de novo synthesis. These metabolic alterations can be exploited by therapy, which aims to improve treatment outcome and decrease relapse and reoccurrence. One clinically utilised strategy targeting AA dependency is the use of asparaginase in the treatment of acute lymphoblastic leukaemia (ALL), which results in a depletion of exogenous asparagine and subsequent cancer cell death. Examples of other successful strategies include the exploitation of arginine deiminase and methioninase, nutrient restriction of methionine and the inhibition of glutaminase. In this review, we summarise these treatment strategies into three promising avenues: AA restriction, enzymatic depletion and inhibition of metabolism. This review provides an insight into the complexity of metabolism in cancer, whilst highlighting these three current research avenues that have support in both preclinical and clinical settings.

## Introduction

Cancer is a disease at the cellular level that results from oncogenic mutations that lead to altered cellular metabolism, promoting tumorigenesis (Pavlova and Thompson 2016). Malignant cells often demonstrate characteristic changes in their metabolism including increased rates of glutaminolysis and fatty acid synthesis, in addition to an increased uptake of glucose (Fadaka et al. 2017). These metabolic changes can expose vulnerabilities which are specific to malignant cells, such as an increased metabolic demand and inefficient adenosine triphosphate generation (Vettore et al. 2019). Literature is now highlighting the importance of AAs as metabolites and metabolic regulators in supporting oncogenesis, despite their primary role in protein synthesis (Ananieva and Wilkinson 2018; Li and Zhang 2016). Due to increased metabolic demands, an abundant supply of nutrients such as glucose and AAs are important for cancer cells to sustain their proliferative drive (Vettore et al. 2019). Therefore, nutrient deprivation strategies have been proposed as an alternative therapy for cancer treatment.

**Fig. 1:**
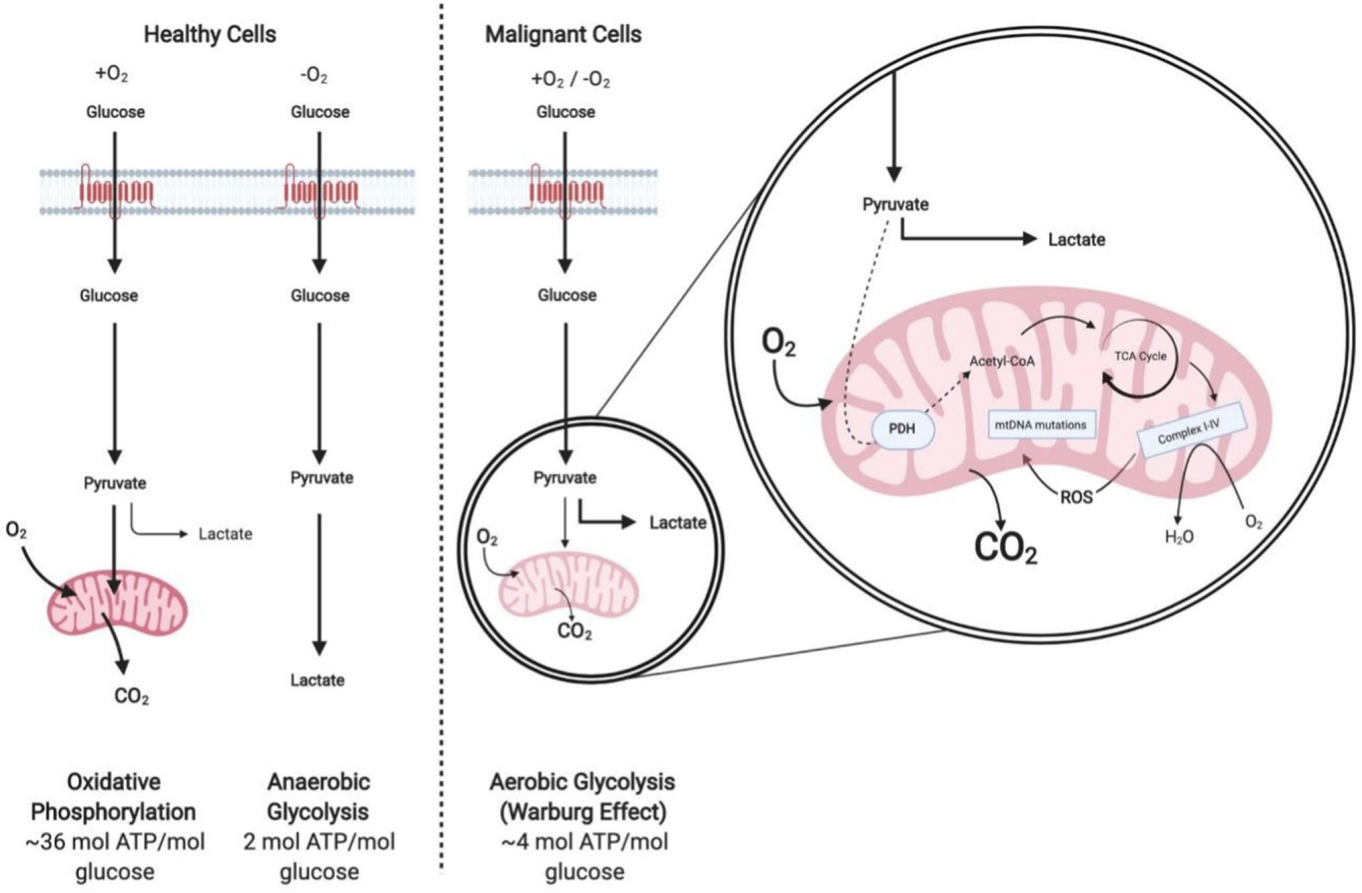
A representation of the differences between oxidative phosphorylation, anaerobic glycolysis and aerobic glycolysis (Warburg effect). In the presence of oxygen, healthy cells metabolise glucose to pyruvate via glycolysis. Pyruvate is oxidised within the mitochondria to produce CO_2_ during oxidative phosphorylation. In the absence of oxygen, cells generate lactate from pyruvate. This allows glycolysis to continue with reduced ATP production in comparison to oxidative phosphorylation. Alternatively, malignant cells undergo aerobic glycolysis regardless of oxygen status. Whilst mitochondria remain functional, minimal oxidative phosphorylation can take place. Abbreviations: *ATP* adenosine triphosphate; *Complex I-IV* mitochondrial respiratory chain complexes I-IV; *mtDNA* mitochondrial DNA; *PDH* pyruvate dehydrogenase; *ROS* reactive oxygen species; *TCA* tricarboxylic acid. Adapted in Biorender from Vander Heiden et al. (2009); Kim and Dang (2006)

An increased rate of glycolysis, and therefore glucose consumption, is exhibited by many malignant cells and is referred to as the Warburg effect (Fig. 1; Warburg 1956), highlighting glucose consumption as a potential target for therapeutic intervention. However, glucose restriction leads to systemic toxicity due to the effects of deprivation on non-malignant cells (Jeon et al. 2016). Unlike glucose, the ability to synthesise non-essential amino acids (NEAAs) de novo is commonly maintained in non-malignant cells yet is lost in malignancy. This ensures non-malignant cells are unaffected by specific AA restrictions, resulting in the emergence of AA restriction as a viable therapeutic strategy. Like glucose, there are major differences in the uptake and metabolism of several AAs in malignant versus non-malignant cells (Jain et al. 2012). It is believed that AAs, rather than glucose, account for the majority of the carbon biomass in proliferating cells, highlighting a significant demand for AAs (Hosios et al. 2016).

Amino acid restriction has already proven to be clinically effective in the treatment of ALL, with the use of asparaginase. Asparaginase is a bacterial derived enzyme that depletes serum levels of the NEAA, asparagine. Asparaginase represents one of the first examples of a therapeutic approach targeting AA metabolism by exploiting AA auxotrophy in cancer, and has resulted in notable improvement of paediatric ALL outcomes. Asparaginase treatment is one of the most successful metabolism-targeting therapies to date, and its success has prompted further studies looking into the effects of exploiting AA metabolism in malignant cells (Vettore et al. 2019). A number of strategies have been proposed to exploit AA metabolism and the increased demand for nutrients in malignant cells. These strategies include dietary restriction of AAs, enzymatic depletion of exogenous AAs and inhibition of enzymatic AA metabolism. All of these methods highlight specific vulnerabilities and potential avenues for cancer therapy. This review will summarise key studies relating to the targeting of AA metabolism in cancer therapy; including ALL, melanoma and hepatocellular carcinoma (Table 1).

**Table 1:** Summary of key evidence identified in this review

Treatment mechanism	Model type	Preclinical/clinical evidence	References
Enzymatic depletion			
Asparaginase	Clinical use in ALL	Mortality rates in patients aged 0–24 years have progressively decreased by 82% from 1971 to 2016 since the introduction of asparaginase	Cancer Research UK (2018)
Arginine deiminase	Human metastatic melanoma clinical trials	Phase 2 clinical trials predicted an increase in median survival of 8 months (7 vs 15 months) in stage IV metastatic melanoma patients	Ascierto et al. (2005)
Recombinant methioninase	Pancreatic cancer and melanoma patient-derived orthotopic xenograft nude-mouse models	When treated with methioninase, mice demonstrated a reduced tumour volume in both pancreatic (694 vs 201 mm^2^) and melanoma (3755 vs 858 mm^2^) cancer models	Kawaguchi et al. (2018)
Nutritional restriction			
Branched chain amino acids	Clinical use in MSUD	Potential activity against cancers that are dependent on BCAAs, yet evidence of its efficacy is lacking	N/A
Methionine	Azoxymethane-induced colon carcinogenesis male F344 rat model	A methionine restricted diet resulted in a reduction of colonic cell proliferation by 12% compared to controls and reduced preneoplastic aberrant crypt foci	Komninou et al. (2006)
Ketogenic diet	Luciferase-tagged VM-M3 mouse models of metastatic cancer	The ketogenic diet was associated with a reduced tumour growth and increased mean survival (31.2 vs 48.9 days)	Poff et al. (2013)
Enzymatic inhibition			
Alanine aminotransferase 2	ALT2 knockdown in human NSCLC cell lines	Approximately a 40% reduction in relative cell density in ALT2 knockdown NSCLC cells compared to wildtype	Hodakoski et al. (2019)
Gabapentin	Human glioma cell lines	Approximately, a 56% concentration-dependent reduction in glioma proliferation when treated with gabapentin	Tönjes et al. (2013)
CB-839 (Telaglenastat)	Human advanced or metastatic renal cell carcinoma clinical trials	A phase 2 clinical trial demonstrated an increase in median progression free survival of 1.9 months (3.8 vs 1.9 months) when treated with everolimus and CB-839 compared to the control	Motzer et al. (2019)

The table contains key treatment mechanisms defined into the three potential avenues of targeting AA metabolism for cancer treatment; enzymatic depletion, nutritional restriction and enzymatic inhibition. The table details both preclinical and clinical evidence in addition to the experimental model type used, whilst summarising the key data in support of targeting AA metabolism in cancer

*AA* amino acid; *ALL* acute lymphoblastic leukaemia; *ALT* alanine aminotransferase; *BCAAs* branched chain amino acids; *MSUD* maple syrup urine disease; *NSCLC* non-small cell lung carcinoma

## Enzymatic depletion of amino acids

Treatment consisting of AA metabolising enzymes has clinically demonstrated anti-proliferative efficacy against cancer, notably ALL. Non-malignant cells have an innate ability to synthesise NEAAs, yet malignant cells often have underlying genetic mutations that result in a loss of this ability. This results in many cancers becoming auxotrophic for certain NEAAs and therefore tumours must rely on exogenous sources of these AAs for growth and survival (Vettore et al. 2019). In the example of ALL, one of the hallmarks of the disease is a lymphoblast deficiency in asparagine synthetase. High rates of protein synthesis and a poor ability to synthesise asparagine drives ALL lymphoblasts into becoming dependent on the extracellular availability of asparagine (Chiu et al. 2019).

Administration of asparaginase catalyses the hydrolysis of asparagine in the serum to aspartate and ammonia, resulting in decreased serum asparagine levels and growth inhibition or death of leukemic cells (Cachumba et al. 2016). The ability of non-malignant cells to continue to synthesise asparagine allows the resulting low levels of serum asparagine to disrupt leukemic cell viability without disturbing non-malignant cells (Ali et al. 2016). Since the introduction of asparaginase in the 1960s for the treatment of ALL, mortality rates in patients aged 0–24 years has progressively decreased by 82% between 1971 and 2016 (Cancer Research UK 2018). The success of asparaginase therapy has prompted the development of additional therapeutics designed to exploit cancer AA dependencies. These metabolic dependencies are described in many cancers, with common deficiencies observed in argininosuccinate synthetase (Patil et al. 2016), and glutamine synthetase (Bolzoni et al. 2016; Chiu et al. 2019; Furusawa et al. 2018).

Arginine auxotrophy has been successfully exploited utilising the microbial derived enzyme, arginine deiminase, which catalyses the conversion of arginine to its precursor citrulline. Clinical trials of arginine deiminase in humans have demonstrated efficacy in both hepatocellular carcinomas and melanomas (Ascierto et al. 2005; Glazer et al. 2010). Initial studies demonstrated a significant reduction in serum arginine levels following arginine deiminase treatment, associated with an increased median survival of 8 months in metastatic melanoma patients compared to controls (Ascierto et al. 2005). Comparatively, advanced hepatocellular carcinoma patients exhibited an overall median survival of 11.4 months (vs 10.7 months), demonstrating an increased efficacy compared to the first-line treatment, sorafenib (Glazer et al. 2010; Llovet et al. 2008).

Recently, arginine deiminase has demonstrated efficacy in a number of alternative cancers including small-cell lung cancer (SCLC) and acute myeloid leukaemia (AML). Kelly et al. (2012) demonstrated that SCLC cells frequently lack expression of argininosuccinate synthetase and treatment with arginine deiminase induced metabolic stress and subsequent autophagy, which progressed to apoptosis in up to 16% of cancer cells. In addition, arginine deiminase treatment in SCLC xenograft mouse models induced a significant dose-dependent reduction in tumour growth compared to controls. Miraki-Moud et al. (2015) demonstrated similar results in AML cells in vivo, finding that arginine deprivation induced by arginine deiminase significantly reduced the percentage of AML cells by more than 50% in the bone marrow of mouse models. Utilising the same models, arginine deiminase in combination with the chemotherapeutic agent cytarabine (commonly used to treat AML) demonstrated improved efficacy than cytarabine alone (Miraki-Moud et al. 2015).

Another example supported by recent evidence is the enzymatic depletion of the essential AA, methionine. Cancer cell proliferation appears dependent on exogenous methionine and is known as methionine dependence or the Hoffman effect (Hoffman 2015; Kaiser 2020). Hoffman and Erbe (1976) demonstrated that malignant cells were able to synthesise high levels of methionine endogenously, yet still insufficient levels to maintain cancer growth. Stern and Hoffman (1984) noted that an overall increase in transmethylation reactions within malignant cells is likely the basis of this methionine-dependence. This alteration within malignant cells has resulted in methionine addiction being recognised as a fundamental hallmark of cancer and oncogenic transformation (Booher et al. 2012; Chello and Bertino 1973; Coalson et al. 1982; Halpern et al. 1974; Hoffman 2015; Hoffman and Erbe 1976; Hoffman and Jacobsen 1980; Hoffman et al. 1979; Mecham et al. 1983; Stern et al.^1984^; Yamamoto et al. 2020).

First reported in 1973, methioninase was initially isolated from *Clostridium sporogenes* and successfully inhibited the growth of Walker carcinosarcoma 256 implanted in male Wistar rats (Kreis and Hession 1973). Recently, with the use of patient-derived orthotopic xenograft (PDOX) mouse models of cancer, Kawaguchi et al. (2018) demonstrated that pancreatic cancer and melanoma exhibited methionine dependency. It was demonstrated that mice treated with recombinant methioninase exhibited a reduced tumour volume in both pancreatic (694 vs 201 mm^2^) and melanoma (3755 vs 858 mm^2^) cancer models (Kawaguchi et al. 2018). This work proposed that the recombinant methioninase decreased serum methionine levels, which in turn resulted in an inadequate methionine supply to the tumours, leading to a reduction in tumour volume.

Following the success of methioninase in preclinical models, studies have highlighted the potential of both intravenous and oral recombinant methioninase in human clinical trials. In a phase 1 clinical trial assessing metastatic breast cancer patients, plasma methionine levels decreased significantly when administered with 20,000 units of recombinant methioninase (Tan et al. 1996). However, this trial did not assess anti-tumour activity. More recently, Han et al. (2020) investigated the use of oral recombinant methioninase as a supplement in advanced cancers. This study demonstrated an approximate 70% decrease in prostate-specific antigen in a patient with bone-metastatic prostate cancer following a twice daily dose of 250 units of methioninase over a 3-month period. Furthermore, the patient’s haemoglobin increased from 7.4 to 8.7 g/dl during the trial, suggesting increased bone functionality following treatment. Whilst the Han et al. (2020) study has demonstrated a potentially safe and effective treatment to reduce circulating methionine, double blind clinical trials are needed to assess efficacy regarding patient survival.

Taken together, the data from these studies supports the enzymatic depletion of AAs as a successful cancer therapy. Although this review focusses on the enzymes with the widest clinical support, there are whole families of enzymes that have yet to be explored. However, whilst there is promise in these therapies many patients experience a hypersensitivity reaction to the infused protein which limits the treatments use (Battistel et al. 2020; Zarei et al. 2019), highlighting a need for orally available treatments (Han et al. 2020). This also leaves opportunities for other mechanisms of AA depletion, such as dietary restriction. The dietary restriction of phenylalanine and branched chain amino acids (BCAAs) are already performed in the clinical setting of phenylketonuria and maple syrup urine disease, respectively (Blackburn et al. 2017).

## Nutritional restriction of amino acids

Otto Warburg first noted changes in the metabolism of glucose within malignant cells in order to support rapid cell proliferation, growth and survival (Warburg 1956). Regardless of oxygen status, malignant cells often produce energy by glycolysis followed by lactic acid fermentation (Fig. 1; Li et al. 2015). This altered metabolism has been exploited clinically through the use of fluorodeoxyglucose positron emission tomography, enabling the detection of malignant tumours (Hsu and Sabatini 2008). Due to the high rate of glucose consumption by malignant cells, glucose deprivation is an attractive therapeutic avenue in many cancers. Numerous studies have demonstrated significantly lower glucose concentrations in malignant cells compared to corresponding non-malignant tissues (Rocha et al. 2015; Urasaki et al. 2012; Ziebart et al. 2011), with average reductions of up to 46% reported in lung tumours (Rocha et al. 2015).

Malignant cells can even adapt to glucose poor conditions by adopting alternative metabolic pathways to support growth. It has been demonstrated in conditions of glucose deprivation, malignant cells can synthesise glucose de novo from non-carbohydrate sources (gluconeogenesis) (Grasmann et al. 2019). Hodakoski et al. (2019) identified that the catabolism of alanine by alanine aminotransferase 2 (ALT2) to pyruvate, was critical for the survival of non-small cell lung carcinoma (NSCLC) cells during glucose starvation. After knockdown of *ALT2*, cells were significantly more sensitive to glucose withdrawal compared to wildtype cells, which were rescued when supplemented with pyruvate. Increased *ALT2* expression has also been reported in human breast cancer and demonstrated a positive correlation with tumour grade and proliferation (Cao et al. 2017). This research highlights the significant role of glucogenic AAs to feed gluconeogenesis in glucose deprived environments (Cao et al. 2017; Hodakoski et al. 2019). However, as 19 out of 22 AAs are considered glucogenic, restriction does not seem practical in a clinical setting.

A common example of dietary cancer therapy is the ketogenic diet, providing a fat-rich, low carbohydrate diet. The rationale is to reduce circulating glucose levels and induce ketosis. This promotes energy starvation in malignant cells that are unable to utilise ketone bodies, whilst non-malignant cells adapt (Klement 2017; Weber et al. 2018). The ketogenic diet has been demonstrated to decrease cancer progression within mouse models of metastatic cancer, slowing tumour growth and increasing mean survival by 56.7% (31.2 vs 48.9 days) (Poff et al. 2013). Zhou et al. (2007) compared a standard diet to a ketogenic diet where the major difference between these diets was the ketogenic ratio of 4:1 (fat: carbohydrate and protein) when following the ketogenic diet. Intracerebral tumour growth of both the CT-2A and U87-MG tumours were reduced by approximately 65% and 35%, respectively. This was associated with improved survival in the ketogenic diet group when compared to control mice. Further to these studies, Rieger et al. (2014) used mouse models to observe the effects of a ketogenic diet in combination with bevacizumab, a drug designed to block vascular endothelial growth factor (Byrne et al. 2005). The combination of both bevacizumab and a ketogenic diet resulted in a 42% reduction in tumour volume when compared to mice receiving bevacizumab alone (13.8 mm^3^ vs 23.9 mm^3^), which was reflected in an increased survival. Despite the promising preclinical data, human studies have not shown consistent clinical improvement. However, many human studies are limited by factors including low patient numbers, poor adherence, lack of randomisation and the absence of a control group. These factors are controlled in preclinical studies utilising animal models, which may be the reason that preclinical success was not replicated in the human trials.

Increasing evidence has highlighted that BCAAs, leucine, isoleucine and valine, are required for malignant growth and act as a source of energy in many cancers (Ananieva and Wilkinson 2018). Recent evidence has demonstrated that the key enzymes in BCAA metabolism: the branched chain aminotransferase (BCAT) and the branched chain keto acid dehydrogenase complex are overexpressed in many cancers and correlate with poorer survival, chemotherapeutic resistance, and enhanced cancer cell proliferation, migration and invasion (Conway et al. 2016; Hattori et al. 2017; Mayers et al. 2016; Tönjes et al. 2013; Zheng et al. 2016). Increased expression of BCAT and increased BCAA uptake was observed in mouse NSCLC tumours, in which leucine uptake was over threefold higher than control lung tissue. In NSCLC tumours, leucine uptake contributed to DNA and NEAA synthesis, important for tumour growth, while pancreatic ductal adenocarcinoma (PDAC) tumours demonstrated decreased BCAA uptake and little utilisation (Mayers et al. 2016). In contrast, Dey et al. (2017) demonstrated that BCAT2 overexpression in PDAC cells positively correlated with the aggressive growth in PDAC tumours driven by chr18q21 chromosomal deletion, suggesting (conversely) PDAC tumours are dependent on BCAAs.

It is apparent that BCAA metabolism in cancer is complex. The long-term dietary restriction of all 3 BCAAs is required for maple syrup urine disease treatment and has demonstrated a successful clinical outcome for an otherwise fatal disease (Blackburn et al. 2017). Utilising dietary BCAA restriction in cancer therapy could inhibit the varying carcinogenic pathways that require BCAAs or its metabolites. However, current clinical data assessing dietary interventions targeting cancer are lacking, despite promising pre-clinical data. For example, Komninou et al. (2006) assessed the dietary restriction of methionine on an azoxymethane-induced colon carcinogenesis rat model. The formation of preneoplastic aberrant crypt foci in the colon was reduced in rats fed on a methionine restricted diet, with a 12% reduction in colonic cell proliferation compared to controls. However, dietary restriction is often complicated due to the complex AA content of food. The isolation of a single AA whilst maintaining a varied and nutritious diet is difficult but is possible when following a diet of medical origin. However, it may be more clinically manageable to instead target the metabolism of specific AAs, rather than restricting them.

## Enzymatic inhibition of amino acid metabolism

A number of pharmacological inhibitors have been extensively researched to inhibit the function of key enzymes involved in AA metabolism, thus disrupting cancer progression. The BCAT enzyme has been identified in having a central role in the pathogenesis of a number of cancers (Hattori et al. 2017; Mayers et al. 2016; Tönjes et al. 2013), with knockdown of the *BCAT* gene demonstrating reduced proliferation of cancer cells both in vitro and in vivo (Hattori et al. 2017; Tönjes et al. 2013). The medication gabapentin, originally designed as a gamma‐aminobutyric acid‐mimetic, has demonstrated both anticonvulsive and analgesic effects. Although the mechanism of gabapentin remains unclear, its main molecular target involves the inhibition of voltage-gated calcium channels at dorsal root ganglion neurons, which contributes to its pain attenuating effects (Kukkar et al. 2013). However, evidence has described gabapentin as an inhibitor of BCAT1. Structural analysis has demonstrated gabapentin acts as an analogue of leucine and can competitively inhibit the transaminase activity of BCAT1 (Goto et al. 2005). In vitro studies have demonstrated that inhibition of BCAT1 by gabapentin dose-dependently disrupts clonogenic growth in chronic myeloid leukaemic cells and promotes intracellular BCAA accumulation (Hattori et al. 2017). Supporting this, following treatment with gabapentin levels of valine, leucine and isoleucine increased by factors of 1.83, 2.18 and 2.32, respectively, in gliomas that harbour wildtype *isocitrate dehydrogenase 1* (Tönjes et al. 2013). This reduced BCAT activity was associated with reduced glutamate release and led to reduced proliferation and invasiveness in vitro (Tönjes et al. 2013).

Not all studies have confirmed that the anti-proliferative effects of gabapentin on tumour cells are dependent of BCAT1 inhibition. Grankvist et al. (2018) demonstrated that at 10 mM, gabapentin significantly reduced the growth of colon cancer cells by approximately 50% at 96 h, despite little BCAT1 expression and BCAT2 as the major isoform. Additionally, the cells were supplemented with branched chain keto acids which failed to recover cell growth in the presence of gabapentin, demonstrating that inhibition of cancer cell growth was not due to inhibition of BCAT2. The mechanism by which gabapentin suppresses cancer cell growth in these cells remains unclear. However, the diverse anti-cancer activity of gabapentin highlights it as a candidate for treatment against cancers with different metabolic phenotypes. For example, malignancies that lack BCAT activity and have little dependency on BCAAs or those that rely on BCAT2 activity rather than BCAT1 (Dey et al. 2017; Grankvist et al. 2018; Mayers et al. 2016).

**Fig. 2:**
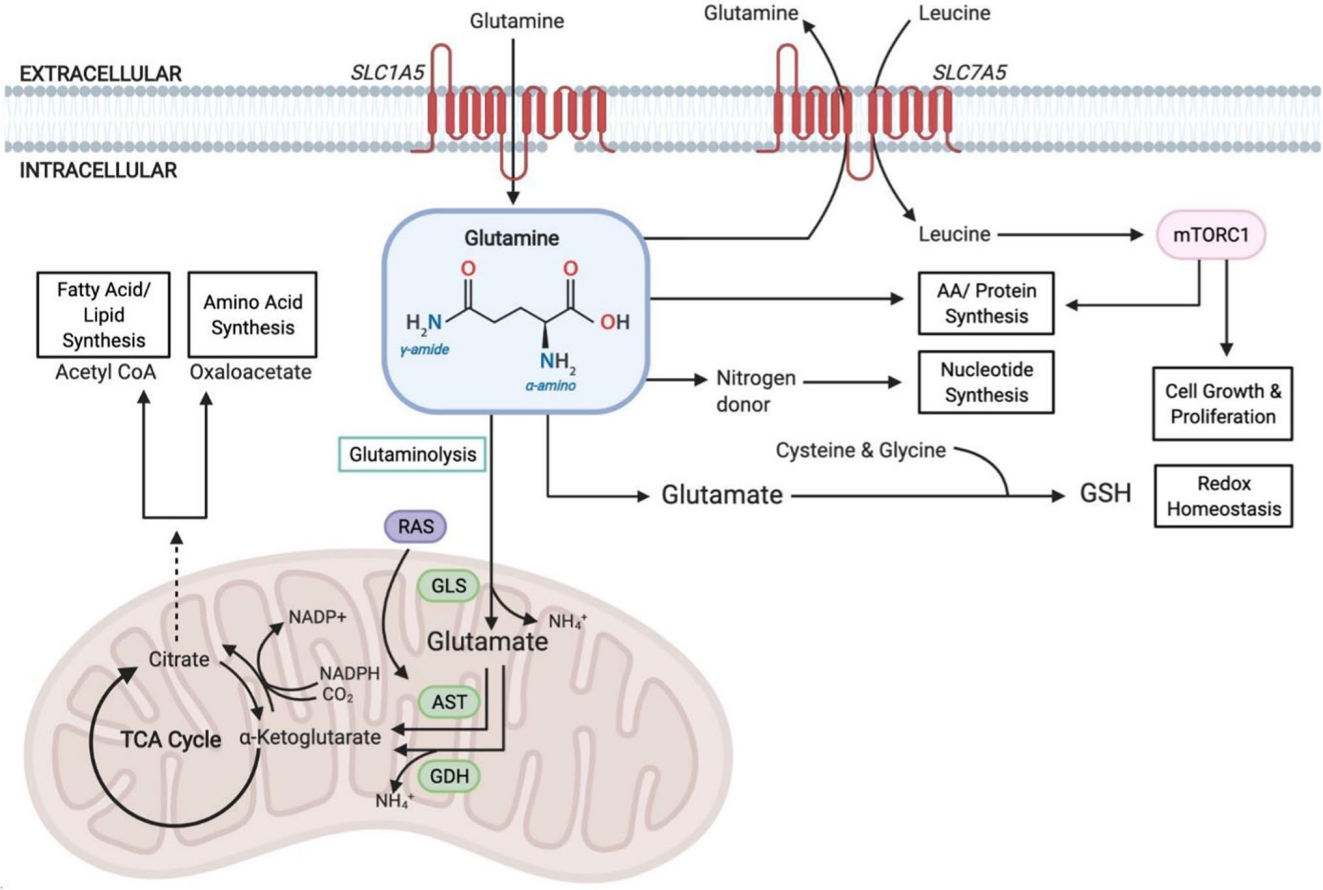
The role of glutamine in cancer. Glutamine enables rapidly proliferating cells to meet increased energy demands in addition to its utilisation in protein synthesis. Glutamine enters the cell via the amino acid transporter SLC1A5 and is converted to glutamate in the mitochondria via a deamination reaction catalysed by GLS. Glutamate is further converted to the TCA cycle intermediate α-ketoglutarate. α-ketoglutarate is a critical metabolite involved in both ATP production and replenishing TCA cycle intermediates (anaplerosis). Cytosolic glutamate is critical for maintaining redox homeostasis through the production of GSH which protects against oxidative stress. Glutamine efflux via the SLC7A5 amino acid transporter allows leucine to enter the cell and activate mTORC1-mediated cell growth. Abbreviations: *AST* aspartate transaminase; *ATP* adenosine triphosphate; *GDH* glutamate dehydrogenase; *GLS* glutaminase; *GSH* S-glutathione; *mTORC1* mammalian target of rapamycin complex 1; *NADP* ± nicotinamide adenine dinucleotide phosphate; *RAS* rat sarcoma GTPase; *SLC1A5* solute carrier family 1 member 5; *SLC7A5* solute carrier family 7 member 5; *TCA* tricarboxylic acid. Adapted in Biorender from Choi and Park (2018); Hensley et al. (2013); Martinez-Outschoorn et al. (2017)

In the 1950s, a dependence on an abundant supply of exogenous glutamine was noted in mammalian cell lines (Eagle 1955). Glutamine metabolism contributes to a number of diverse roles which support the ability of malignant cells to meet increased energy demands and continuously grow. This includes energy generation, cell proliferation, maintaining redox homeostasis and the synthesis of NEAAs, fatty acids and nucleotides (Fig. 2; Martinez-Outschoorn et al. 2017). However, recent studies have demonstrated that glutamine requirements are heterogeneous amongst different cell lines and that the tissue of origin, genetics and the tumour microenvironment all impact the utilisation of glutamine within the cancer cell (Cluntun et al. 2017). As a NEAA, glutamine is synthesised de novo by the enzyme glutamine synthetase (Tardito et al. 2015). However, some malignancies rely on an exogenous supply of glutamine due to a low expression of glutamine synthetase (Cluntun et al. 2017). Therefore, in the absence or inhibition of glutaminase, malignancies which rely on energy from an exogenous supply of glutamine will be unable to utilise nutrients from this source.

There are a number of approaches to target glutamine metabolism including blocking cellular uptake of glutamine, depletion of glutamine in the blood and inhibition of enzymes involved in glutamine synthesis and catabolism, all of which provide potential avenues for cancer therapy (Lukey et al. 2013; Martinez-Outschoorn et al. 2017). Targeting glutaminase function utilising small molecules or gene knockdown approaches has produced promising preclinical results in many malignancies such as NSCLC and B cell lymphoma (Le et al. 2012; Van den Heuvel et al. 2012). Glutaminase inhibitors have demonstrated success in both preclinical and clinical studies. Telaglenastat (CB-839) is a potent and selective inhibitor of both splice variants of glutaminase, which has exhibited antiproliferative effects on triple negative breast cancer cell lines and xenograft models (Gross et al. 2014). In a patient-derived triple negative breast cancer xenograft, Gross et al. (2014) demonstrated a 61% inhibition of tumour growth with telaglenastat treatment alone. Furthermore, treatment with a combination of telaglenastat and paclitaxel demonstrated a 100% tumour inhibition relative to controls.

Phase 1 clinical trials have demonstrated the success of telaglenastat as a well-tolerated monotherapy in patients with multiple myeloma and lymphoma through the successful inhibition of glutaminase in tumours (Vogl et al. 2015). Vogl et al. (2015) also highlighted the success of telaglenastat in combination with pomalidomide/ dexamethasone, supporting the clinical use of telaglenastat for relapsed multiple myeloma. Further phase 1 studies also demonstrated the success of telaglenastat as a monotherapy in addition to studying combinations with other drugs such as everolimus in patients with renal cell carcinoma (Meric-Bernstam et al. 2016). As a result of the success demonstrated in phase 1 clinical trials, the work has progressed into phase 2 with one active phase 2 trial comparing telaglenastat in combination with everolimus to a placebo with everolimus in advanced or metastatic renal cell carcinoma. The randomised, double-blind, placebo-controlled phase 2 trial is approaching completion (National Institute of Health 2017). However, initial results from the trial are promising, with a median progression-free survival of 3.8 months when patients were treated with telaglenastat and everolimus, compared to 1.9 months for those who received the placebo and everolimus (Motzer et al. 2019). This promising initial data, along with the progression of other studies to phase 2 clinical trials, provides support for the use of telaglenastat as a successful glutaminase inhibitor.

## Conclusion

The extensive mutational heterogeneity of cancer highlights a growing need for personalised medicine. Therapies currently exist to target the many pathways associated with tumour progression. Yet many patients suffer due to the toxicity of these treatments, in addition to cancer cell adaptations which results in chemoresistance. Research needs to identify novel therapeutic avenues to tackle the resistant forms of these cancers or provide a less aggressive alternative to current therapies in patients not deemed fit to treat. Amino acids are required for a number of vital processes and many malignant cells exhibit genetic abnormalities that compromise their ability to obtain or utilise an adequate supply of the AAs required for tumorigenesis. Nutrient restriction, enzymatic depletion and enzymatic inhibition of AA metabolism have all demonstrated success in preclinical and clinical models, supporting these therapies as viable strategies for cancer treatment (Table 1). These alternative approaches have the potential to revolutionise standard clinical practice and could transform relapse and reoccurrence statistics for many cancers. However, initial promising results need to be supported by double-blind clinical trials. The research summarised in this review promises success in the underlying principle of AA restriction as an effective non-genotoxic therapy, that can be utilised as both a monotherapy or combination therapy.

## Data Availability

Not applicable.

## Abbreviations

AA: Amino acid
ALL: Acute lymphoblastic leukaemia
ALT: Alanine aminotransferase
AML: Acute myeloid leukaemia
BCAA: Branched chain amino acid
BCAT: Branched chain aminotransferase
NEAA: Non-essential amino acid
NSCLC: Non-small cell lung carcinoma
PDAC: Pancreatic ductal adenocarcinoma
PDOX: Patient-derived orthotopic xenograft
SCLC: Small-cell lung cancer
